# *Trypanosoma Cruzi* Lineages Shape Macrophage Cytokine Profiles in Single and Mixed Infections

**DOI:** 10.1111/pim.70029

**Published:** 2025-09-12

**Authors:** Cecília Luiza Pereira, Giovana Maria Salmazi de Carvalho, Laura Eduarda Miranda da Silva, Anna Clara Azevedo Silveira, Elida Cristina Monteiro de Oliveira, Claudio Vieira da Silva

**Affiliations:** ^1^ Universidade Federal de Uberlândia Uberlândia Brazil

**Keywords:** chagas disease, coinfection, cruziome, cytokines, macrophages, *trypanosoma cruzi*

## Abstract

Chagas disease, caused by *Trypanosoma cruzi*, exhibits a wide clinical spectrum, which is influenced by the parasite's extensive genetic diversity. Growing evidence indicates that human infections are often multiclonal, involving a dynamic population of parasite clones, collectively termed the “cruziome”. The immunological consequences of these mixed infections, particularly at the initial host–parasite interface, remain poorly characterised. This study aimed to investigate how single versus co‐infection with phylogenetically distinct *T. cruzi* strains modulates the innate immune response of murine macrophages. RAW 264.7 macrophages were infected with *T. cruzi* strains from Discrete Typing Unit (DTU) I (G strain) or DTU II (Y strain), either individually or in combination (co‐infection). Assays for cellular invasion and intracellular multiplication were performed. The production of key cytokines (IL‐1β, IL‐18, IL‐6, IL‐10, IL‐12) and nitric oxide (NO) was quantified at 24 to 96 h post‐infection. The Y strain displayed significantly higher invasion and replication rates and induced a potent pro‐inflammatory response, characterised by elevated levels of IL‐1β, IL‐18, IL‐12, and NO. The G strain elicited a more regulatory profile, with a progressive increase in IL‐10 production at later time points and lower levels of inflammatory mediators. Co‐infection resulted in a distinct, hybrid immune profile, marked by intermediate levels of both pro‐inflammatory and regulatory cytokines and a moderated NO output. Co‐infection with phylogenetically distinct *T. cruzi* strains generates a unique immunomodulatory environment that is not merely an additive effect of the individual strains. These findings provide in vitro evidence supporting the hypothesis that the composition of the infecting parasite population shapes the host immune response from the earliest stages of infection. This balanced interplay between pro‐inflammatory and regulatory signals may contribute to the clinical heterogeneity observed in Chagas disease and underscores the need to consider parasite diversity in pathogenic and therapeutic studies.

## 1 Introduction

Chagas disease, a chronic parasitic infection caused by the protozoan *Trypanosoma cruzi*, remains a major public health issue in Latin America with increasing global reach. The disease is characterised by remarkable clinical heterogeneity, ranging from lifelong asymptomatic forms to severe and often fatal cardiac or digestive manifestations [1, 2, 3]. This variability is influenced by a combination of factors, including host genetics and route of infection, but is thought to be significantly driven by the parasite's vast genetic diversity. *T. cruzi* is classified into seven Discrete Typing Units (DTUs): TcI to TcVI and Tcbat. These lineages exhibit distinct geographical distributions and are associated with different clinical outcomes. For instance, TcI is predominant in northern South America and is often linked to asymptomatic or milder forms of the disease, whereas TcII is prevalent in the Southern Cone and is frequently associated with severe chronic Chagas cardiomyopathy. This genetic variability translates into profound phenotypic differences, including variations in virulence, tissue tropism, and the ability to modulate the host immune response [4]. The Y strain (TcII) is a well‐established model of a virulent parasite, known for its high infectivity and its capacity to induce a strong pro‐inflammatory response characterised by IL‐12, TNF‐α, and IFN‐γ production. In contrast, the G strain (TcI) typically exhibits lower infectivity and is associated with a more regulatory immune response that favours IL‐10 production, a profile that may facilitate long‐term parasite persistence [5, 6, 7]. While many experimental studies have historically utilised single parasite populations for clarity, this approach may not fully represent the complexity of natural infections. It is important to note that many laboratory ‘strains’ of *T. cruzi* are, in fact, isolates that may contain heterogeneous parasite populations rather than true, single cell‐derived clones. Recent advances in molecular diagnostics have challenged the monoclonal infection paradigm for Chagas disease. It is now understood that infections are frequently multiclonal, with a single host harbouring a complex and dynamic mixture of different *T. cruzi* clones. This co‐existing population of clones and their variants has been termed the “cruziome”. Co‐infections with multiple DTUs have been reported in human patients, mammalian reservoirs, and insect vectors, suggesting that this is the norm rather than the exception in endemic settings [7, 8]. The interactions within this mixed parasite population can profoundly alter infection dynamics and pathogenesis in ways that are not predictable from single‐strain infections [8]. Macrophages play a pivotal role in the early host response to *T. cruzi* . They function as both effector cells that eliminate the parasite and permissive host cells for its replication. Upon activation, macrophages can polarise into a classically activated (M1) phenotype, producing inflammatory mediators and nitric oxide (NO) to control pathogen proliferation, or an alternatively activated (M2) phenotype, associated with immune regulation and tissue repair [9, 10, 11]. The balance between these activation states is critical in determining the outcome of the infection. While the general roles of macrophages are known, how the initial interaction with a mixed parasite population, a simplified “cruziome”, shapes their activation state is not well understood [8, 9, 10, 11]. In this context, the present study was designed to investigate how *T. cruzi* strains from distinct phylogenetic lineages (DTU I and DTU II) modulate the response of murine macrophages, both in single and co‐infection conditions. By characterising the cytokine production profile and nitric oxide synthesis, this work aims to elucidate how the genetic diversity of the parasite shapes the innate immune response and to explore the potential implications of these interactions for the progression of Chagas disease.

## 2 Materials and Methods

### 2.1 Cell Culture and Parasite Maintenance

The murine macrophage cell line RAW 264.7 was cultured in RPMI 1640 medium (Gibco, USA) supplemented with 10% fetal bovine serum (FBS), 100 U/mL penicillin, and 100 μg/mL streptomycin. Cells were maintained at 37°C in a humidified atmosphere containing 5% CO₂. *T cruzi* strains Y (DTU II) and G (DTU I) are well‐characterised. G strain, isolated from an opossum in the Amazon region, was personally provided by Nobuko Yoshida (UNIFESP) and originally from Mena Barreto. Y strain was isolated from a human blood sample in 1953 by Vicente Amato Neto. Their DTU classifications were confirmed by previous molecular typing [12]. They were maintained by weekly passages in LLC‐MK2 cells cultured in DMEM supplemented with 10% FBS under the same incubation conditions. Trypomastigote forms used in the experiments were obtained from the supernatants of infected LLC‐MK2 cell cultures, and their viability was confirmed by motility assessment using light microscopy prior to each experiment.

### 2.2 Cell Invasion and Intracellular Multiplication Assays

RAW 264.7 macrophages were seeded in 24‐well plates containing 13 mm glass coverslips at a density of 1 × 10^5^ cells per well and incubated for 24 h to allow for cell adhesion. For the invasion assay, macrophages were infected with trypomastigotes from either Y or G strains at a multiplicity of infection (MOI) of 5 for 2 h. A multiplicity of infection (MOI) of 5 was chosen for invasion and multiplication assays to allow for clear visualisation and quantification of individual intracellular parasites without oversaturating the host cells. For cytokine and nitric oxide quantification, a higher MOI of 10 was used to ensure a robust and measurable macrophage activation and secretory response, consistent with established protocols for in vitro immune analysis. The 2‐h time point specifically quantifies internalised parasites, as a rigorous washing step was performed to remove any non‐internalised, attached trypomastigotes. Following incubation, non‐internalised parasites were removed by washing with 1X PBS. Cells were then fixed with methanol and stained using a Panoptic staining kit (Laborclin, Brazil) for microscopic quantification of intracellular amastigotes. To assess intracellular multiplication, cells were infected under the same conditions (MOI 5, 2 h), and after removal of extracellular parasites, they were incubated for different time points (24‐, 48‐, 72‐, and 96‐h post‐infection). At each point, cells were fixed, stained, and the number of intracellular amastigotes was quantified to evaluate the replicative capacity of the parasites. For all microscopic analyses, at least 300 cells were counted per coverslip in triplicate.

### 2.3 Cytokine Quantification

For cytokine analysis, RAW 264.7 macrophages were plated and infected with *T. cruzi* strains Y, G, or both (co‐infection) at an MOI of 10 for 2 h. In the co‐infection group, an MOI of 5 for each strain was used. A group of uninfected cells served as a control. Supernatants were collected at 24 and 96 h post‐infection for cytokine measurement. Levels of IL‐1β and IL‐18 were quantified using Invitrogen ELISA kits (Thermo Fisher Scientific, USA), while IL‐10, IL‐12, and IL‐6 were measured using BD OptEIA ELISA kits (BD Biosciences, USA), according to the manufacturers' instructions. Absorbance readings were performed at 450 nm using a microplate reader.

### 2.4 Nitric Oxide Quantification

Nitric oxide (NO) production was assessed using the same infection protocol applied for cytokine analysis. Supernatants were collected at 24 and 96 h post‐infection. Nitrite concentration, a stable indicator of NO production, was determined using the Griess reagent system (Promega, USA). Absorbance was measured at 570 nm using a microplate spectrophotometer.

### 2.5 Statistical Analysis

All experiments were performed in triplicate and independently repeated at least three times. Data are presented as mean ± standard deviation (SD). Statistical comparisons between experimental groups were performed using one‐way analysis of variance (ANOVA) followed by Tukey's post hoc test. A *p* < 0.05 was considered statistically significant.

## 3 Results

### 3.1 Y Strain Exhibits Higher Infectivity and Replicative Capacity in Macrophages

**FIGURE 1:**
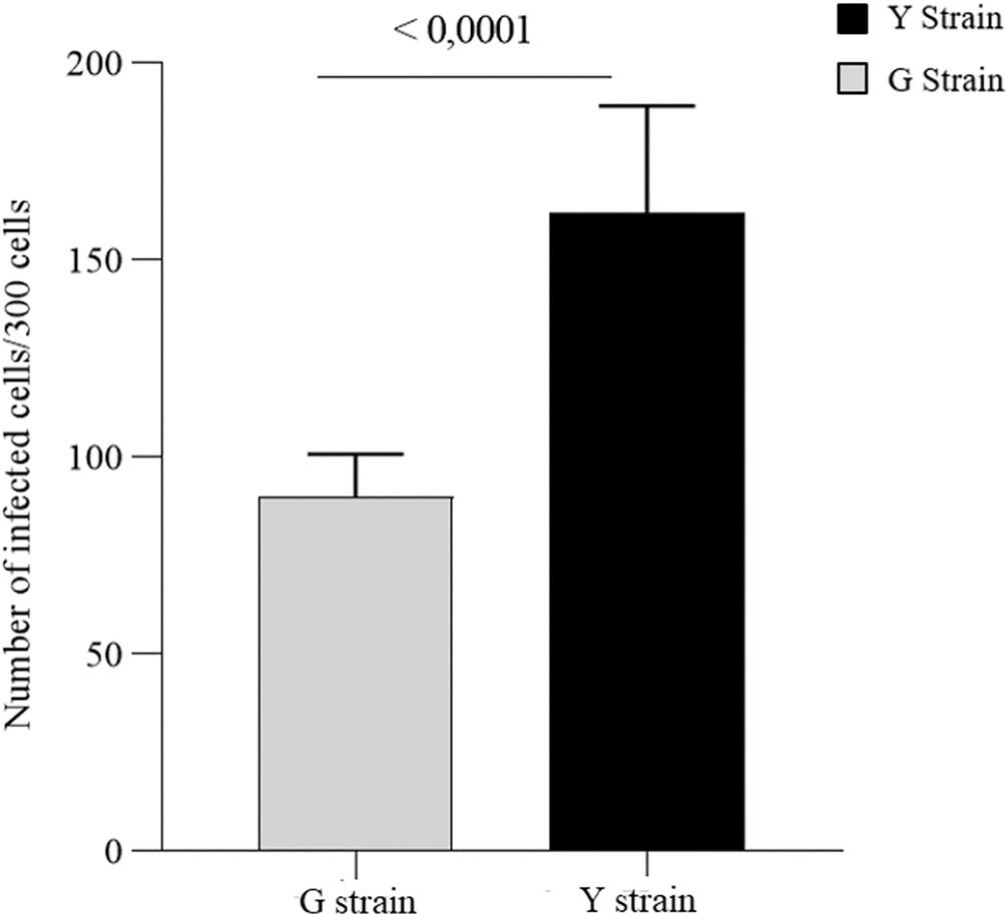
*Trypanosoma cruzi* invasion in RAW 264.7 macrophages. Bars represent the mean ± standard deviation of the number of intracellular parasites per 100 cells. Groups: Cells infected with Y strain and G strain. Data is representative of three independent experiments performed in triplicate. Statistical significance was determined by a t‐test, with *p* < 0.0001.

**FIGURE 2:**
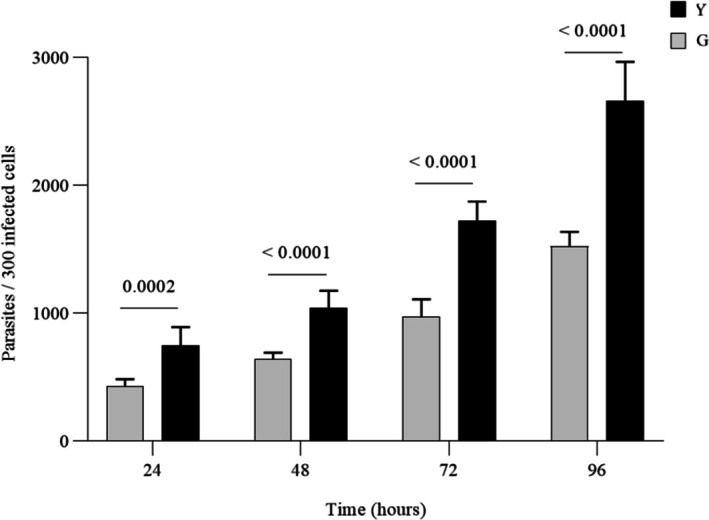
Intracellular multiplication of *Trypanosoma cruzi* strains Y and G in RAW 264.7 macrophages. The total number of intracellular amastigotes was quantified in 300 infected cells at 24‐, 48‐, 72‐, and 96‐h post‐infection (hpi). Data are representative of three independent experiments performed in triplicate and are expressed as mean ± SD. Statistical significance was determined by two‐way ANOVA followed by Sidak's post hoc test. *p* < 0.01, p < 0.0001.

**FIGURE 3:**
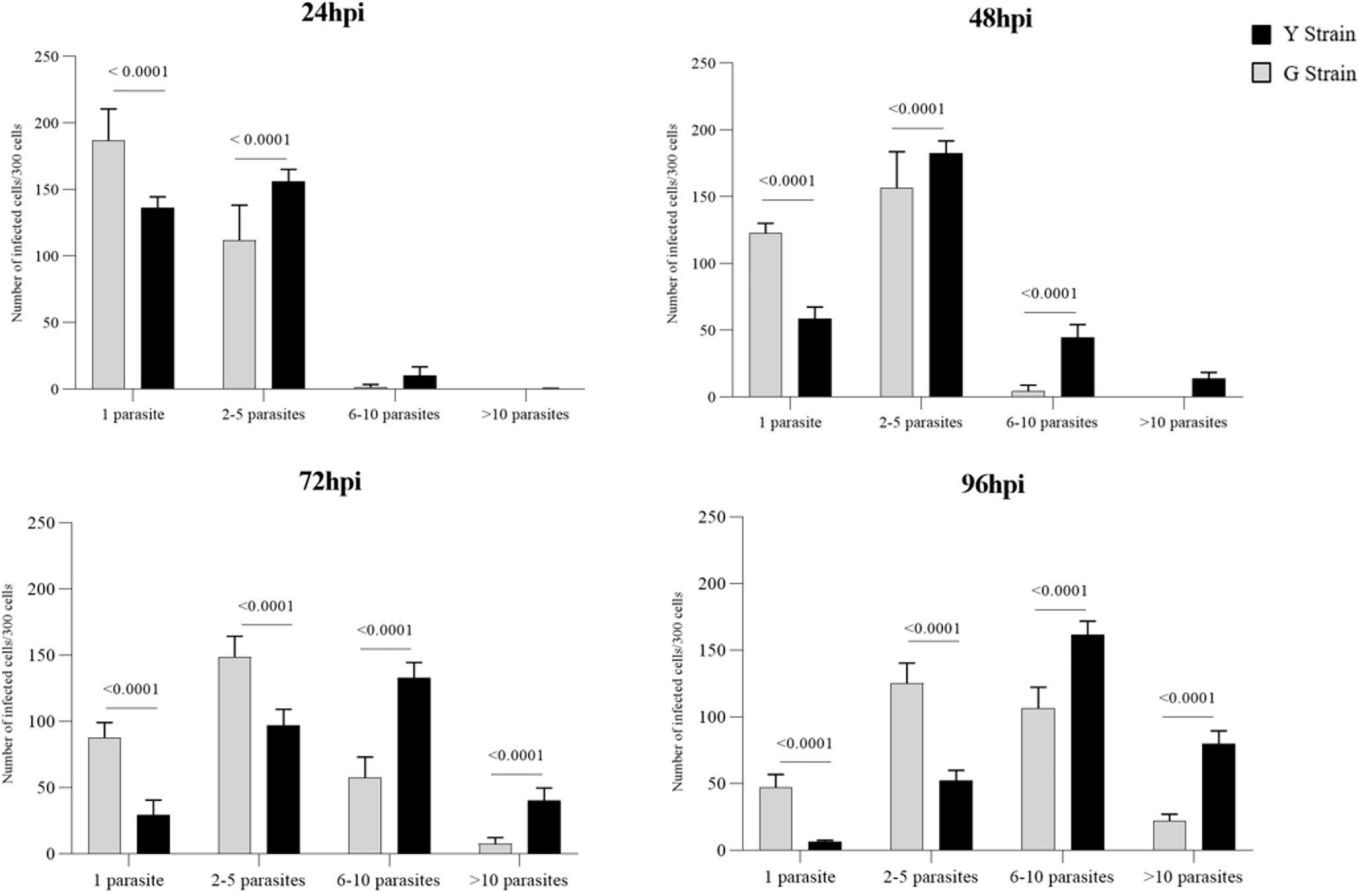
Distribution of intracellular parasite load in RAW 264.7 macrophages infected with *Trypanosoma cruzi* strains Y and G at 24‐, 48‐, 72‐, and 96‐h post‐infection. Intracellular parasites were grouped into ranges (1, 2–5, 6–10, and > 10 parasites per cell), based on counts from 300 infected cells per group. Data are representative of three independent experiments performed in triplicate.

Analysis of the invasion rate revealed significant differences in the infectivity of the Y and G strains in RAW 264.7 macrophages (Figure 1). After 2 h of infection, the Y strain demonstrated greater efficiency in cell invasion, infecting approximately 165 out of 300 analysed cells (an infection rate of ~55%), whereas the G strain infected about 100 out of 300 cells (an infection rate of ~33%) (*p* < 0.0001). This suggests that the Y strain has a higher capacity for adhesion and entry into host cells. The kinetics of intracellular multiplication were evaluated over 96 h (Figure 2). While both strains showed a progressive increase in parasite load over time, the Y strain exhibited a significantly higher rate of intracellular replication at all evaluated time points compared to the G strain (*p* = 0.0002 at 24 h; *p* < 0.0001 at 48, 72, and 96 h). This pattern indicates that the Y strain not only invades host cells more efficiently but also sustains rapid and continuous intracellular replication (Figure 3).

### 3.2 Infection With Distinct *T. cruzi* Strains Induces Divergent Cytokine Profiles

**FIGURE 4:**
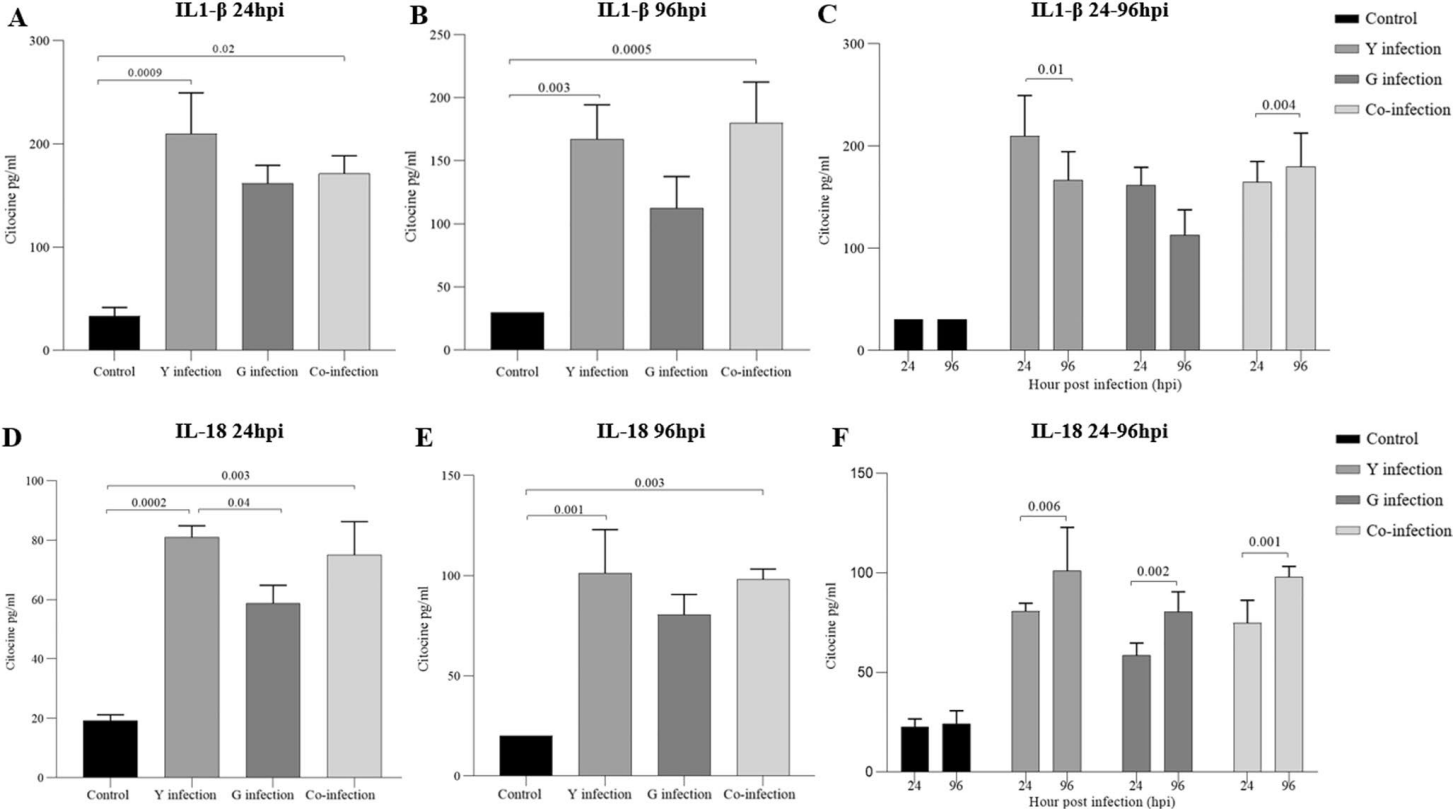
Production of IL‐1β (A–C) and IL‐18 (D–F) by RAW 264.7 macrophages at 24 h and 96 h post‐infection with *Trypanosoma cruzi*. Groups: Control (black), Y strain, G strain, and coinfection. Both cytokines showed significant increases at 24 hpi, particularly in the Y strain group, with partial reductions at 96 hpi. Data are expressed as mean ± SD from three independent experiments performed in triplicate. Statistical significance (*p* < 0.05) was determined by one‐way ANOVA followed by Tukey's post hoc test.

**FIGURE 5:**
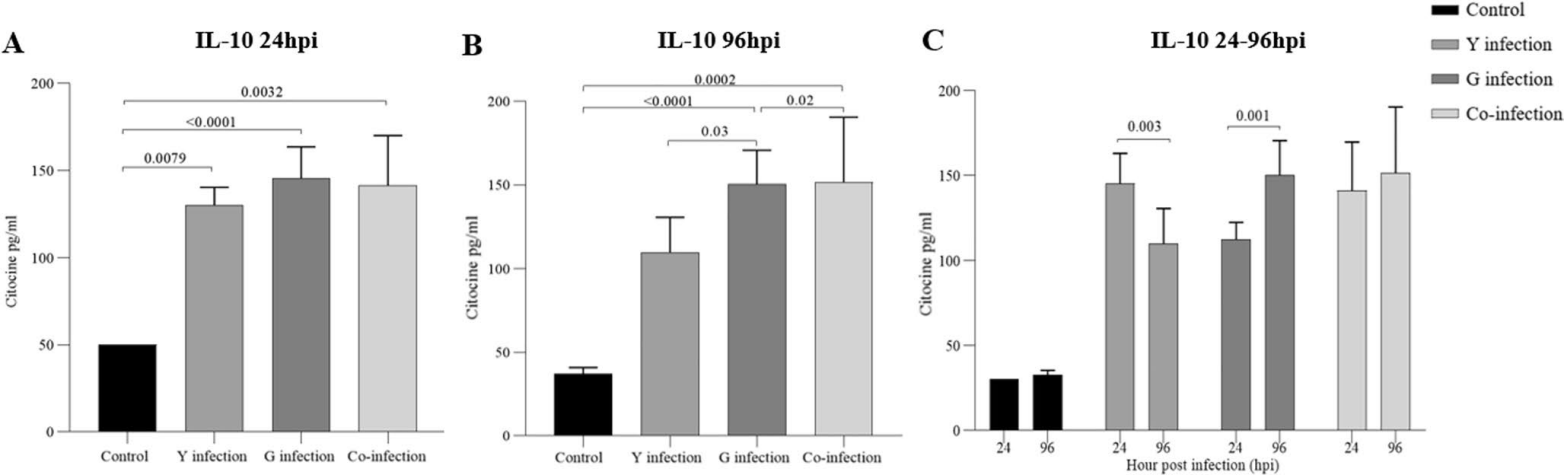
IL‐10 is produced by RAW 264.7 macrophages at 24 h and 96 h post‐infection with *Trypanosoma cruzi*. Groups: Control, Y strain, G strain, and coinfection. A progressive increase in IL‐10 was observed in the G strain group at 96 hpi, while a reduction was noted in the Y strain group compared to 24 hpi. Data are expressed as mean ± SD from three independent experiments performed in triplicate. Statistical significance (*p* < 0.05) was determined by one‐way ANOVA followed by Tukey's post hoc test.

**FIGURE 6:**
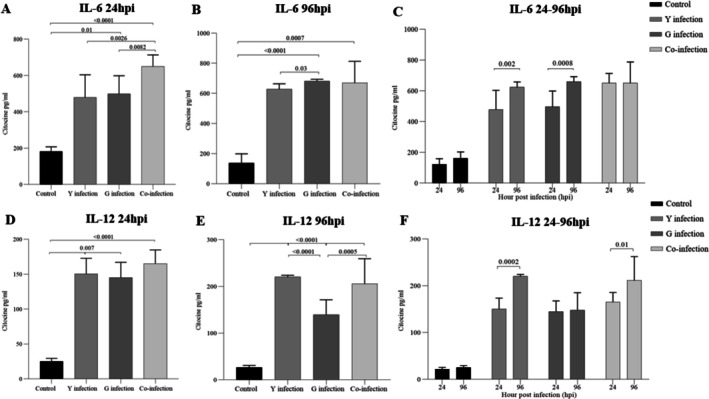
IL‐6 and IL‐12 are produced by RAW 264.7 macrophages at 24 h and 96 h post‐infection with *Trypanosoma cruzi*. Groups: Control, Y strain, G strain, and coinfection. Cytokine levels were significantly elevated in all infected groups compared to the control, with variations depending on the strain and infection time. Data are expressed as mean ± SD from three independent experiments performed in triplicate. Statistical significance (*p* < 0.05) was determined by one‐way ANOVA followed by Tukey's post hoc test.

To investigate the immunomodulatory effects of the different infection conditions, cytokine levels were measured in the macrophage supernatants. The Y strain induced a potent pro‐inflammatory response (Figures 4, 6). At both 24 and 96 h post‐infection, levels of IL‐1β, IL‐18, and IL‐12 were significantly higher in the Y‐infected group compared to all other groups. IL‐6 levels were also strongly elevated by the Y strain. In contrast, the G strain induced a more regulatory profile. While there were modest increases in pro‐inflammatory cytokines compared to the control, they were significantly lower than those induced by the Y strain. Notably, the G strain‐infected group showed a significant and progressive increase in the production of the regulatory cytokine IL‐10 at 96 h post‐infection (*p* < 0.01 compared to 24 h) (Figure 5).

### 3.3 Co‐Infection Results in a Hybrid Immunological Profile

The response of macrophages to co‐infection was unique and not simply an additive effect. The levels of pro‐inflammatory cytokines IL‐1β, IL‐18, and IL‐12 were intermediate, falling between the high levels induced by Y strain and the lower levels induced by G strain (Figures 4, 6). Similarly, IL‐10 production in the co‐infection group was maintained at a moderate level, higher than the late response of Y strain but not reaching the sustained increase seen with G strain alone (Figure 5).

### 3.4 Nitric Oxide Production Correlates With the Inflammatory Profile

**FIGURE 7:**
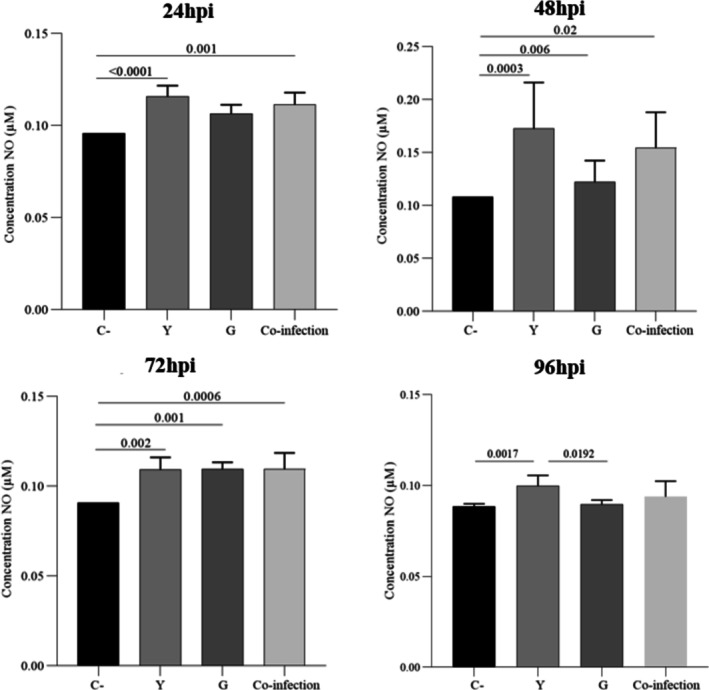
Nitric oxide (NO) is produced by RAW 264.7 macrophages at 24 h, 48 h, 72 h, and 96 h post‐infection with *Trypanosoma cruzi*. Experimental groups: Control, Y strain, G strain, and coinfection with Y + G. NO levels were measured using the Griess reaction. All infected groups showed a significant increase in NO production, particularly the Y strain group at 48 hpi. Data are expressed as mean ± SD from three independent experiments performed in triplicate. Statistical significance (*p* < 0.05) was determined by one‐way ANOVA followed by Tukey's post hoc test.

Nitric oxide production mirrored the cytokine profiles (Figure 7). Infection with Y strain resulted in the most robust induction of NO, which was significantly elevated at all time points. G strain infection led to a more modest but still significant increase in NO compared to the uninfected control. In the co‐infection group, NO production was at an intermediate level, consistent with the moderated inflammatory response observed.

## 4 Discussion

This study investigated the macrophage response to single and mixed infections with phylogenetically distinct *T. cruzi* strains, providing an in vitro model to explore the immunological consequences of multiclonal infections. Our findings demonstrate that the interaction between different parasite lineages generates a unique immunomodulatory landscape that is not a simple summation of the individual responses. This result lends support to the emerging concept of the “cruziome,” where the collective parasite population within a host dictates the nature of the immune response and, potentially, the clinical outcome of the disease [8]. The highly virulent Y strain (DTU II) behaved as expected, inducing a classic M1 macrophage activation profile. Its superior capacity for invasion and replication was coupled with the robust production of pro‐inflammatory cytokines (IL‐1β, IL‐18, IL‐12) and nitric oxide. This aggressive phenotype is consistent with reports linking TcII parasites to more severe pathology [9, 10, 11, 13, 14, 15]. This type of potent, early inflammatory response, while essential for controlling initial parasitemia, can also drive immunopathology and tissue damage characteristic of chronic Chagas disease if not properly regulated [14, 15]. Conversely, the G strain (DTU I) induced a more restrained and regulatory phenotype. Its lower infectivity and replication, combined with a delayed but sustained production of the anti‐inflammatory cytokine IL‐10, suggest a profile that favours parasite persistence over acute host inflammation [15]. This is consistent with the association of TcI parasites with wild reservoirs and milder, indeterminate forms of human Chagas disease, reflecting an evolutionary adaptation that promotes long‐term host survival and transmission. The reduced pro‐inflammatory signalling may allow the parasite to evade complete clearance, establishing a chronic infection [16]. The most significant finding of this study arose from the co‐infection condition. The resulting immune profile was not merely an average of the two single‐strain infections but a distinct, hybrid state [17, 18]. The intermediate levels of both pro‐inflammatory (IL‐1β, IL‐12, NO) and regulatory (IL‐10) mediators suggest a complex interplay and mutual modulation between the two parasite strains. For instance, IL‐12 production in the co‐infection group was approximately 35%–45% lower than that induced by the Y strain alone, while IL‐10 levels were significantly elevated compared to the negligible amounts seen with the Y strain at 96 h. It is plausible that the Y strain drives the initial inflammatory activation, while the G strain concurrently provides a regulatory “brake” that tempers this response over time. This dynamic balance creates a unique immunological environment, highlighting that the infecting parasite consortium functions as an interactive system rather than a simple collection of independent entities [8]. This finding provides an experimental basis for observations in mice, where co‐infection interfered with the host immune response and altered pathological outcomes compared to single infections. Our results also align with vivo studies that show different DTUs can colonise distinct tissues and elicit varied immune responses, which could be further complicated during a mixed infection [19, 20, 21, 22]. These in vitro results have important implications for understanding the clinical variability of Chagas disease. The specific composition of a patient's infecting parasite population could be a key determinant of their clinical trajectory. A population dominated by highly inflammatory clones, like Y strain, might predispose an individual to progressive cardiac or digestive disease. In contrast, a population composed of more regulatory clones, like G strain, or a balanced, hybrid mix as modelled by our co‐infection, could lead to the long‐term, asymptomatic indeterminate form of the disease. In this scenario, the host immune system achieves a state of equilibrium, controlling parasite replication without causing excessive tissue damage. The concept of “harmful” versus “neutral” cruziomes, as proposed by Carlier et al., finds direct support in our cellular model. This study has limitations. It is an in vitro model using a single macrophage cell line and two representative parasite strains. The complex in vivo environment involves a multitude of other immune cells and factors that contribute to the overall response. However, this focused approach provides clear, foundational evidence of the direct modulatory effects of co‐infection at the cellular level [13, 23]. Future studies should expand upon these findings using in vivo co‐infection models to assess how these initial innate responses shape the subsequent adaptive immune response and long‐term tissue pathology. Furthermore, correlating the molecular characterisation of patient “cruziomes” with their immunological profiles and clinical status will be essential for validating these concepts in human Chagas disease.

## 5 Conclusion

In conclusion, this study demonstrates that single infections with *T. cruzi* strains Y (DTU II) and G (DTU I) elicit divergent, predictable pro‐inflammatory and regulatory macrophage responses, respectively. Critically, co‐infection with both strains results in a distinct, hybrid immunological profile characterised by a moderated and balanced production of inflammatory and regulatory mediators. This suggests a complex, modulatory interplay between parasite lineages at the earliest stages of infection. These findings underscore the importance of considering the dynamics of multiclonal infections to fully understand the variable immunopathogenesis of Chagas disease and to develop more nuanced therapeutic strategies.

## Author Contributions

**Cecília Luiza Pereira:** conceptualization, methodology, investigation, formal analysis, Writing – original draft, writing – review and editing, visualisation, data curation. **Giovana Maria Salmazi de Carvalho:** conceptualization, methodology, investigation, validation, visualisation. **Laura Eduarda Miranda da Silva:** data analysis, writing – review and editing. **Anna Clara Azevedo Silveira:** data analysis, writing – review and editing. **Elida Cristina Monteiro de Oliveira:** data analysis, writing – review and editing. **Claudio Vieira da Silva:** conceptualization, supervision, project administration, writing, review and editing.

## Disclosure

The authors have nothing to report.

## Conflicts of Interest

The authors declare no conflicts of interest.

## Peer Review

The peer review history for this article is available at https://www.webofscience.com/api/gateway/wos/peer‐review/10.1111/pim.70029.

## Data Availability

The data that support the findings of this study are available from the corresponding author upon reasonable request.

## References

[1] World Health Organization , “Chagas Disease (American Trypanosomiasis),” 2023.

[2] C. Bern , “Chagas' Disease,” New England Journal of Medicine 373 (2015): 456–466. 26222561 10.1056/NEJMra1410150

[3] A. Rassi , A. Rassi , and J. A. Marin‐Neto , “Chagas Disease,” Lancet 375 (2010): 1388–1402. 20399979 10.1016/S0140-6736(10)60061-X

[4] M. M. A. Silvestrini , G. D. Alessio , B. E. D. Frias , et al., “New Insights Into *Trypanosoma Cruzi* Genetic Diversity, and Its Influence on Parasite Biology and Clinical Outcomes,” Frontiers in Immunology 15 (2024): 431. 10.3389/fimmu.2024.1342431 PMC11035809 38655255

[5] J. C. S. Aliberti , M. A. G. Cardoso , G. A. Martins , R. T. Gazzinelli , L. Q. Vieira , and J. S. Silva , “Interleukin‐12 Mediates Resistance to *Trypanosoma Cruzi* in Mice and Is Produced by Murine Macrophages in Response to Live Trypomastigotes,” Infection and Immunity 64 (1996): 1961–1967. 8675294 10.1128/iai.64.6.1961-1967.1996 PMC174023

[6] T. A. Da Costa , M. V. Silva , M. T. Mendes , et al., “Immunomodulation by *Trypanosoma Cruzi*: Toward Understanding the Association of Dendritic Cells With Infecting TcI and TcII Populations,” Journal of Immunology Research 2014 (2014): 962047. 25371910 10.1155/2014/962047 PMC4211313

[7] C. M. Rodrigues , H. M. S. Valadares , A. F. Francisco , et al., “Coinfection With Different *Trypanosoma Cruzi* Strains Interferes With the Host Immune Response to Infection,” PLoS Neglected Tropical Diseases 4 (2010): e846. 20967289 10.1371/journal.pntd.0000846 PMC2953483

[8] Y. Carlier , E. Dumonteil , C. Herrera , et al., “Coinfection by Multiple *Trypanosoma Cruzi* Clones: A New Perspective on Host‐Parasite Relationship With Consequences for Pathogenesis and Management of Chagas Disease,” Microbiology and Molecular Biology Reviews 89 (2025): 224. 10.1128/mmbr.00242-24 PMC12188747 40116484

[9] L. A. Messenger , M. A. Miles , and C. Bern , “Between a Bug and a Hard Place: *Trypanosoma Cruzi* Genetic Diversity and the Clinical Outcomes of Chagas Disease,” Expert Review of Anti‐Infective Therapy 13 (2015): 995–1029. 26162928 10.1586/14787210.2015.1056158 PMC4784490

[10] G. Macaluso , F. Grippi , S. Di Bella , et al., “A Review on the Immunological Response Against *Trypanosoma Cruzi* ,” Pathogens 12 (2023): 282. 36839554 10.3390/pathogens12020282 PMC9964664

[11] Í. Rodrigues‐dos‐Santos , M. F. Melo , L. de Castro , et al., “Exploring the Parasite Load and Molecular Diversity of *Trypanosoma Cruzi* in Patients With Chronic Chagas Disease From Different Regions of Brazil,” PLoS Neglected Tropical Diseases 12 (2018): e0006939. 30418976 10.1371/journal.pntd.0006939 PMC6258420

[12] B. Zingales and D. C. Bartholomeu , “ *Trypanosoma Cruzi* Genetic Diversity: Impact on Transmission Cycles and Chagas Disease,” Memórias do Instituto Oswaldo Cruz 117 (2022): e210193. 35544857 10.1590/0074-02760210193 PMC9088421

[13] S. J. Koo , I. H. Chowdhury , B. Szczesny , X. Wan , and N. J. Garg , “Macrophages Promote Oxidative Metabolism to Drive Nitric Oxide Generation in Response to *Trypanosoma Cruzi* ,” Infection and Immunity 84 (2016): 3527–3541. 27698021 10.1128/IAI.00809-16 PMC5116729

[14] N. S. Vellozo , T. C. Matos‐Silva , and M. F. Lopes , “Immunopathogenesis in *Trypanosoma Cruzi* Infection: A Role for Suppressed Macrophages and Apoptotic Cells,” Frontiers in Immunology 14 (2023): 1244071. 37662946 10.3389/fimmu.2023.1244071 PMC10469960

[15] R. T. Gazzinelli , I. P. Oswald , S. Hieny , S. L. James , and A. Sher , “The Microbicidal Activity of Interferon‐y‐Treated Macrophages Against *Trypanosoma Cruzi* Involves an L‐Arginine‐Dependent, Nitrogen Oxide‐Mediated Mechanism Inhibitable by Interleukin‐10 and Transforming Growth Factor‐β,” European Journal of Immunology 22 (1992): 2501–2506. 1396957 10.1002/eji.1830221006

[16] B. Zingales , “ *Trypanosoma Cruzi* Genetic Diversity: Something New for Something Known About Chagas Disease Manifestations, Serodiagnosis and Drug Sensitivity,” Acta Tropica 184 (2018): 38–52. 28941731 10.1016/j.actatropica.2017.09.017

[17] L. M. D. Magalhães , L. S. A. Passos , E. Chiari , et al., “Co‐Infection With Distinct *Trypanosoma Cruzi* Strains Induces an Activated Immune Response in Human Monocytes,” Parasite Immunology 41 (2019): e12668. 31494949 10.1111/pim.12668 PMC6800597

[18] L. M. D. Magalhães , K. J. Gollob , B. Zingales , and W. O. Dutra , “Pathogen Diversity, Immunity, and the Fate of Infections: Lessons Learned From *Trypanosoma Cruzi* Human‐Host Interactions,” Lancet Microbe 3 (2022): e711–e722. 36058233 10.1016/S2666-5247(21)00265-2

[19] H. Sales‐Campos , H. B. Kappel , C. P. Andrade , et al., “ *Trypanosoma Cruzi* DTU TcII Presents Higher Blood Parasitism Than DTU TcI in an Experimental Model of Mixed Infection,” Acta Parasitologica 60 (2015): 435–441. 26204180 10.1515/ap-2015-0060

[20] G. B. M. Dias , A. P. Gruendling , S. M. Araújo , M. L. Gomes , and M. J. d. O. Toledo , “Evolution of Infection in Mice Inoculated by the Oral Route With Different Developmental Forms of *Trypanosoma Cruzi* I and II,” Experimental Parasitology 135 (2013): 511–517. 23994765 10.1016/j.exppara.2013.08.013

[21] F. Y. Maeda , T. M. Clemente , S. Macedo , C. Cortez , and N. Yoshida , “Host Cell Invasion and Oral Infection by *Trypanosoma Cruzi* Strains of Genetic Groups TcI and TcIV From Chagasic Patients,” Parasites & Vectors 9 (2016): 156. 27038796 10.1186/s13071-016-1455-z PMC4818890

[22] T. B. D. Queiroga , N. d. S. Pereira , D. D. da Silva , et al., “Virulence of *Trypanosoma Cruzi* Strains Is Related to the Differential Expression of Innate Immune Receptors in the Heart,” Frontiers in Cellular and Infection Microbiology 11 (2021): 696719. 34336720 10.3389/fcimb.2021.696719 PMC8321543

[23] F. R. S. Gutierrez , T. W. P. Mineo , W. R. Pavanelli , P. M. M. Guedes , and J. S. Silva , “The Effects of Nitric Oxide on the Immune System During *Trypanosoma Cruzi* Infection,” Memórias do Instituto Oswaldo Cruz 104, no. Suppl 1 (2009): 236–245. 19753479 10.1590/s0074-02762009000900030

